# Safe and Efficient Extracorporeal Photopheresis in Patients with Pre-Procedure Hematocrit below 26%

**DOI:** 10.1007/s12288-025-02064-1

**Published:** 2025-05-31

**Authors:** Andriana Pavlovich, Faith Matthews, Yamac Akgun

**Affiliations:** 1 Department of Pathology and Laboratory Medicine, Children’s Hospital Los Angeles, Los Angeles, CA USA; 2 University of Southern California Keck School of Medicine, Los Angeles, CA USA

**Keywords:** Extracorporeal Photopheresis, Hematocrit, Graft-versus-Host Disease, Apheresis Safety

## Abstract

**Background:**

Extracorporeal photopheresis (ECP) is widely used for graft-versus-host disease (GVHD), but manufacturer recommendations suggest maintaining a pre-procedure hematocrit (Hct) above 27%. This study evaluates the safety and efficacy of ECP performed at lower Hct levels.

**Methods:**

We retrospectively analyzed 13 ECP procedures in three male patients with GVHD, comparing sessions performed at Hct < 26% to those at Hct > 27%. Procedural parameters, hemodynamic stability, and adverse events were assessed.

**Results:**

All procedures at Hct < 26% were successfully completed without adverse reactions, vascular access issues, or significant hemodynamic changes. Average procedure duration was 97 min at Hct < 26% and 95 min at Hct > 27%, showing no significant impact on efficiency.

**Conclusion:**

ECP at Hct < 26% is safe and effective, reducing the need for transfusion while maintaining procedural integrity. These findings challenge current Hct thresholds and support more flexible patient selection criteria.

## Introduction

Extracorporeal photopheresis (ECP) is a well-established therapeutic apheresis modality that involves the collection of leukocytes, exposure to a photosensitizing agent (8-methoxypsoralen), ultraviolet A irradiation, and reinfusion of the treated cells [1]. ECP is primarily used for immune modulation in conditions such as graft-versus-host disease (GVHD), cutaneous T-cell lymphoma, and certain autoimmune disorders [2]. The mechanism of action includes apoptosis of treated lymphocytes, antigen presentation, and subsequent immunomodulatory effects, making ECP an essential therapy for chronic inflammatory and immune-mediated diseases [3].

GVHD, a common complication of hematopoietic stem cell transplantation, occurs when donor-derived immune cells attack the recipient’s tissues. It manifests as acute or chronic disease affecting multiple organs, including the skin, liver, and gastrointestinal tract [4]. Patients with GVHD frequently suffer from anemia due to a combination of factors, including bone marrow suppression, chronic inflammation, gastrointestinal bleeding, and medication effects [5]. These patients often require long-term ECP therapy, undergoing 2–3 procedures per week for several months or even years [6]. Since transfusions are not always feasible or desirable due to concerns about iron overload, alloimmunization, or transfusion reactions, strictly maintaining a pre-procedure hematocrit (Hct) above 27% presents a significant challenge [7, 8].

Manufacturer guidelines, including those from Therakos, generally recommend maintaining a pre-procedure Hct above 27% for optimal safety and efficiency [8]. However, strict adherence to this threshold may limit access to ECP for anemic patients who could otherwise benefit. This study examines the safety and efficacy of ECP performed on patients with pre-procedure Hct below 26% to assess whether outcomes differ from procedures conducted at higher Hct levels.

## Materials and Methods

### Patient Demographics and Clinical Characteristics

This study retrospectively analyzed 13 ECP procedures performed on 3 patients between May 2023 and March 2025. The study focused on cases where pre-procedure hematocrit was below 26% to evaluate procedural success, hemodynamic stability, and technical adverse events. The same patients also underwent multiple ECP procedures with Hct values above 27%, serving as an internal control. The three patients included in the study ranged in age from 14 to 19 years, all of whom were males. All patients had chronic GVHD requiring long-term ECP therapy, undergoing multiple sessions per week. All patients were between 6 and 12 months post-hematopoietic stem cell transplantation.

### Procedure Details

The procedures were performed using the Therakos Cellex ECP system. Extracorporeal volume (ECV) was calculated using Nadler’s equation for total blood volume (TBV) and manufacturer-specified ECV based on patient hematocrit. Each session lasted between 1.5 and 2 h. In all procedures 1500 ml of blood was processed. Flow rates remained at default manufacturer settings, and no adjustments were necessary for tolerance in low-Hct procedures. Vital signs monitored included heart rate, blood pressure, oxygen saturation, and temperature. A clinically significant change in vital signs was defined as a heart rate variation of > 20 bpm, blood pressure deviation of > 15% from baseline, oxygen saturation drop below 92%, or temperature variation > 1.5 °C. Technical adverse events were defined as any machine malfunctions, alarms, or flow issues that required intervention to continue the procedure. This included interruptions due to extracorporeal volume limits, pressure sensor faults, or anticoagulation-related concerns.

## Results

All 13 procedures performed on patients with a pre-procedure hematocrit of less than 26% were successfully completed without any complications. There were no reported adverse reactions, technical issues, or difficulties in vascular access. Additionally, there were no significant differences in key procedural parameters, including total blood volume processed or procedure duration when compared to procedures conducted with a pre-procedure Hct above 27%. Hemodynamic stability was maintained throughout all sessions, with no significant fluctuations in heart rate, blood pressure, or oxygen saturation before, during, or after the procedures. Importantly, no patients required transfusions, intravenous fluids, or other medical interventions to support their hemodynamic status.

Post-procedure hematocrit levels measured 1 h after the procedure showed a mean decrease of only 0.2%. Furthermore, no machine alarms were triggered due to low Hct, and anticoagulation requirements remained consistent across all procedures. The average procedure duration was 97 min when pre-procedure hematocrit was below 26%, compared to 95 min for the same patients when it was above 27%, showing no clinically significant delay or inefficiency (Table 1).

**Table 1:** ECP procedural data for patients with Pre-Procedure hematocrit below 26%

Patient Number	Weight (kg)	Pre-Procedure Hematocrit (%)	Procedure Length (minutes)	Total Blood Volume Processed (mL)
1	59.3	24.1	95	1500
1	59.3	25.3	100	1500
1	59.7	25.3	105	1500
1	60	25.2	100	1500
2	84.4	26	85	1500
2	85.6	25.6	100	1500
2	86.1	25.5	110	1500
2	86.3	25.3	95	1500
3	70.4	25.5	85	1500
3	67.2	23	95	1500
3	67.2	24.6	100	1500
3	68.1	25.2	90	1500
3	67.6	25.9	100	1500

## Discussion

The findings challenge the necessity of maintaining a pre-Hct > 27% for safe and effective ECP. The absence of adverse events, technical difficulties, or hemodynamic instability in procedures conducted at lower Hct levels suggests that ECP can be safely performed in anemic patients, potentially expanding eligibility for this therapy. Hematocrit, while commonly used to assess anemia, may not accurately reflect total red cell mass, particularly in volume-expanded patients. Since transfusions are not always practical or desirable in patients with chronic anemia due to GVHD, lowering the Hct threshold for ECP can significantly reduce the need for blood transfusions. This, in turn, minimizes the risks associated with transfusions, such as iron overload, alloimmunization, and transfusion-related reactions.

Additionally, deferring ECP due to low Hct may delay treatment and impact clinical outcomes. The ability to proceed with ECP without prior transfusion ensures that patients receive uninterrupted therapy, which is crucial in chronic conditions like GVHD. Furthermore, avoiding unnecessary transfusions may reduce overall healthcare costs and resource utilization in transfusion-dependent patients.

From a technical perspective, our study demonstrated that lower Hct did not impact procedural success or machine performance. No alarms or flow issues were encountered, and anticoagulation requirements remained stable across all sessions. Based on the standard return protocol with full rinseback, no red cell loss was noted in the tubing post-procedures.

Although manufacturer guidelines recommend a pre-procedure hematocrit > 27%, our institutional standard operating procedures do not mandate a specific hematocrit threshold for initiating the procedure. The decision to proceed is made at the discretion of the medical director based on a comprehensive clinical assessment. In all cases, informed consent is obtained, and the procedure is performed under direct physician supervision.

This suggests that the current manufacturer recommendations could be revisited to allow more flexibility in patient selection. These findings suggest that performing ECP on patients with a hematocrit below 26% does not compromise the safety, efficiency, or overall success of the procedure. Patients with significant comorbidities, such as cardiovascular disease, should be carefully selected for this procedure, with individualized risk-benefit assessment and appropriate clinical monitoring, as further investigation is warranted in these populations. In addition, all subjects were male adolescents; generalizability to females or older adults may be limited and warrants further study.

Supporting our findings, Liu et al. [9] also demonstrated that a lower transfusion threshold for ECP in adult GVHD patients did not compromise safety or collection efficiency, further reinforcing that strict hematocrit requirements may be unnecessary. Their study highlighted that maintaining a lower Hct threshold does not negatively impact procedural outcomes, aligning with our observations that ECP can be safely performed below the traditionally recommended 27% hematocrit level.

Future research with a larger cohort could further validate these findings and potentially influence guideline modifications. Establishing a revised threshold for pre-ECP Hct could enhance treatment accessibility for anemic patients without compromising safety or efficacy.

## Conclusion

This study provides evidence that ECP procedures conducted at pre-procedure Hct < 26% are safe and effective, with outcomes comparable to those performed at manufacturer-recommended levels. These findings support reconsideration of rigid Hct thresholds, allowing greater flexibility in patient selection and access to ECP treatment while reducing unnecessary transfusions and associated complications. Lowering the Hct threshold for ECP could help streamline treatment protocols, reduce delays, and improve patient outcomes by ensuring continuous therapy without the need for pre-procedure transfusions. As transfusions carry inherent risks and resource demands, adopting a more flexible approach could have significant clinical and operational benefits.
